# Dietary supplementation with *Cyberlindnera jadinii* improved growth performance, serum biochemical Indices, antioxidant status, and intestinal health in growing raccoon dogs (*Nyctereutes procyonoides*)

**DOI:** 10.3389/fmicb.2022.973384

**Published:** 2022-09-21

**Authors:** Dehui Zhao, Hanlu Liu, Haihua Zhang, Keyuan Liu, Xinyu Zhang, Qian Liu, Yan Wu, Ting Zhang, Qiaoru Zhang

**Affiliations:** ^1^College of Agriculture, Chifeng University, Chifeng, China; ^2^Hebei Key Laboratory of Specialty Animal Germplasm Resources Exploration and Innovation, Hebei Normal University of Science and Technology, Qinhuangdao, China; ^3^Institute of Special Animal and Plant Sciences of Chinese Academy of Agricultural Sciences, Changchun, China

**Keywords:** *Cyberlindnera jadinii* (*Candida utilis*), growth performance, immunity, antioxidant, microbiota, raccoon dogs

## Abstract

This study was conducted to investigate whether different dietary *Cyberlindnera jadinii* levels affect growth performance, serum immunity, antioxidant capacity, and intestinal microbiota in growing raccoon dogs. Forty-five healthy male raccoon dogs were randomly assigned to three treatment groups, with 15 raccoon dogs per group. Each raccoon dog was housed in an individual cage. The raccoon dogs in the three groups were fed diets supplemented with *Cyberlindnera jadinii* at dosages of 0 (N group), 1 × 10^9^ (L group) and 5 × 10^9^ CFU/g (H group). A 7-day pretest period preceded a formal test period of 30 days. The results showed that *Cyberlindnera jadinii* in the L and H groups improved average daily gain (ADG) (*P* < 0.05) and decreased the ratio of feed to weight (F/G) (*P* < 0.05). Serum immunoglobulins A and G levels were increased in the L and H groups compared to the N group (*P* < 0.05). *Cyberlindnera jadinii* in the L and H groups increased serum superoxide dismutase activity (*P* < 0.05), and serum glutathione peroxidase activity was increased in the L group compared to the N group (*P* < 0.05). The relative abundance of Firmicutes and Actinobacteriota were increased, and the relative abundance of Bacteroidota was decreased in the L and H groups compared to the N group (*P* < 0.05). The relative abundance of Proteobacteria and Cyanobacteria was increased in the H group compared to the other two groups (*P* < 0.05). The ratio of Firmicutes to Bacteroidetes in the *Cyberlindnera jadinii* supplementation groups increased compared with the N group (*P* < 0.05). The relative abundance of *Megasphaera* and *Bifidobacterium* were increased, and the relative abundance of *Prevotella* was decreased in the L and H groups compared to the N group (*P* < 0.05). The relative abundance of *Dialister* was increased, while the relative abundance of *Blautia* was decreased in the H group compared to the other two groups (*P* < 0.05). The relative abundance of *Agathobacter* was decreased in the H group compared to the N group (*P* < 0.05). In conclusion, dietary supplementation with *Cyberlindnera jadinii* increased growth performance, serum immunity, antioxidant capacity, and improved intestinal microbiota in growing raccoon dogs. *Cyberlindnera jadinii* can therefore be used as a growth promoter in raccoon dogs.

## Introduction

Adding antibiotics to animal feed can improve growth and feed conversion efficiency, and prevent infection (Gadde et al., 2017). However, the use of antibiotics leads to antibiotic resistance and the destruction of beneficial bacteria in the gut, which pose a potential threat to animal and public health safety. Therefore, the development of effective antibiotic alternatives is a pressing need. Probiotics are an effective and eco-friendly alternative to antibiotics (Zorriehzahra et al., 2016; Alagawany et al., 2018). Among them, yeast is a promising feed additive that may replace frequently used antimicrobial growth promoters (Alizadeh et al., 2016). Research has shown that feeding yeast and its products can modulate growth performance, and gut microbiota, enhance gut development and integrity, support the immune system, and improve nutrient utilization (Gao et al., 2008; Jiang et al., 2015; Bilal et al., 2021).

The yeast *Cyberlindnera jadinii* is a close relative of *Candida utilis*, which is currently used in the food and feed industries (Rupp et al., 2015). *Cyberlindnera jadinii* can produce valuable bioproducts that are an attractive source of biomass enriched in protein and vitamins (Sousa-Silva et al., 2021). Partially replacing conventional protein sources with inactivated *Cyberlindnera jadinii* yeast in for the diet of young pigs is possible without compromising energy and protein metabolism (Cruz et al., 2020). More importantly, feeding *Cyberlindnera jadinii* yeast can improve post-weanling gut homeostasis and result in more robust piglets (Håkenåsen et al., 2020). *Cyberlindnera jadinii* can also improve growth performance, reduce diarrhea rates, improve intestinal health, and increase the diversity and abundance of cecal microbiota in weaned piglets. Therefore, it may be used as antibiotic alternative feed additive in the production of weaned piglets (Yang et al., 2021). However, there is no research data available regarding the use of *Cyberlindnera jadinii* in the diets of canines.

The Ussuri raccoon dog is the most easily bred fur animal and has high economic value. Unlike in herbivores, carnivorous gut microbiota are specialized to degrade protein as an energy source (Muegge et al., 2011). The gut microbiota is a central regulator of host metabolism, and the composition and function of the gut microbiota is dynamic and affected by dietary properties (Schoeler and Caesar, 2019). The effect of *Cyberlindnera jadinii* on the Ussuri raccoon dog has not been studied. Thus, the present study examined the effects of *Cyberlindnera jadinii* supplementation on growth performance, serum immunity, antioxidant status, and gut microbiota of Ussuri raccoon dogs during the growing period. We hypothesized that *Cyberlindnera jadinii* may affect the growth and gut microbiota of raccoon dogs.

## Materials and methods

All procedures involving animals were carried out in accordance with guidelines for animal studies issued by the Chifeng University.

### Fungal strain

*Cyberlindnera jadinii* were preserved by the microbiology laboratory of the Chifeng University. The liquid potato dextrose culture medium (PDB) (Potato extract powder 20 g/L, glucose 20 g/L, distilled water 1 L) was used to resuscitate and passage cultures for 24–48 h at 37°C.

### Experimental design

Forty-five 60 (±5) day-old healthy male raccoon dogs of a similar body weight (1.98 ± 0.15 kg) were randomly assigned to three treatment groups, with 15 replicates per group. *Cyberlindnera jadinii* was either not added to the diet (N group) or was supplemented at 1 × 10^9^ CFU/g (L group) or 5 × 10^9^ CFU/g (H group). The basal diet was formulated based on the management guide of the National Research Council (National Research Council, 1982); the composition and nutrient levels of the basal diet are shown in Table 1. All animals were individually housed in conventional cages (1.0 m × 0.8 m × 0.8 m). The raccoon dogs were fed twice each day at 7:00 and 15:00 and had free access to water. After 7 days of adaptation, the experimental diets were fed for 30 days.

**TABLE 1:** Composition and nutrient levels of basal diets (air-dry basis).

Items	Content (%)	Items	Content (%)
**Ingredients**		**Nutrient** **levels^2^**	
Extruded corn	40.84	Metabolizable energy (MJ/kg)^2^	14.66
Corn	5.11	Crude protein	21.66
Corn germ meal	9.20	Ether extract	9.43
Rice bran meal	3.07	Ash	6.59
Soybean oil	5.62	Calcium	0.96
Soybean meal	14.82	phosphorus	0.72
Distillers dried grains with solubles	9.20	Lysine	1.26
Dried porcine soluble	1.02	Methionine	0.93
Fish meal	1.63	Methionine + Cysteine	1.22
Meat and bone meal	6.54		
Pork plasma protein powder	1.02		
NaCl	0.20		
Lysine	0.46		
Methionine	0.61		
Choline	0.05		
Premix^1^	0.61		
Total	100.00		

^1^The premix provided the following per kg of diets: VA 18 997 IU, VB_1_ 53 mg, VB_2_ 23 mg, VB_6_ 23 mg, VB_12_ 0.08 mg, VD_3_ 2 658 IU, VE 152 mg, VK_3_ 2.3 mg, biotin 0.66 mg, folic acid 1.2 mg, D-pantothenic acid 21 mg, nicotinamide 46 mg, antioxidant 0.6 mg, Cu (as copper sulfate) 31 mg, Fe (as ferrous sulfate) 114 mg, Mn (as manganese sulfate) 60 mg, Zn (as zinc sulfate) 97 mg, I (as calcium iodate) 0.3 mg, Se (as sodium selenite) 0.23 mg.

^2^Metabolizable energy was a calculated value; the other values were measured.

### Sample collection and preparation

Body weight was measured at the beginning and end of the experiment, and the feed intake of each animal was recorded daily. The average daily gain, average daily feed intake and the ratio of feed to gain (F/G) were calculated according to the following formula: Average daily gain (ADG) (g/d) = (final body weight-initial body weight) (g)/days (d); Average daily feed intake (ADFI) (g/d) = total feed intake (g)/days (d); Ratio of feed to gain (F/G) = average daily feed intake (g)/average daily weight gain (g). After the 30-day supplementation, the raccoon dogs were restrained for blood sampling and approximately 5 mL blood was collected in a vacuum tube from the posterior limb vein. Blood was centrifuged at 3,000 × *g* for 15 min at 4°C to isolate serum which was stored at –20°C until analysis. In addition, fresh feces from each raccoon dog was collected on the same day using 5 mL sterile centrifuge tubes, and stored at –80°C for gut microbiota analyses.

### Serum sample analysis

The serum immunoglobulins (Ig)A, IgM, and IgG were measured using ELISA kits (Shanghai Shuangying Biotechnology Co., Ltd., Shanghai, China). Glutathione peroxidase (GSH-Px), superoxide dismutase (SOD), total antioxidant capacity (T-AOC) and maleic dialdehyde (MDA) were determined using diagnostic kits (Nanjing Jiancheng Bioengineering Institute, Nanjing, China).

### Microbiota analysis based on 16S RNA high-throughput sequencing

Six fecal samples from the raccoon dogs in each group were chosen for microbiota analysis. Total bacterial DNA was extracted from approximately 0.25 g of feces using a Qiagen magnetic bead extraction Kit (Qiagen, Valencia, California, USA) according to the manufacturer’s instructions. The primers 341F (5′-CCTAYGGGRBGCASCAG-3′) and 806R (5′-GGACTACNNGGGTATCTAAT-3′) were used to amplify the V3–V4 region of the bacterial 16S rRNA gene. The resultant amplicons were purified using the Thermo Scientific GeneJET Gel Extraction Kit (Thermo Scientific, Belmont, Massachusetts, USA), and then sequenced on an Illumina NovaSeq 6000 platform to produce 250-bp paired-end reads.

The paired-end reads were merged into raw tags using FLASH version 1.2.7 (Magoè and Salzberg, 2011). Quality filtering of the raw tags was strictly filtered (< 30 Phred score) to obtain high-quality clean tags using QIIME (version 1.9.1) (Caporaso et al., 2010; Bokulich et al., 2013). The tags were compared with the SILVA database (version 138), and the chimera of effective tags were identified and removed using the UCHIME algorithm (Edgar et al., 2011; Haas et al., 2011). The Uparse algorithm (Uparse version 7.0.1001) was used to cluster the effective tags from all samples, and the sequences were clustered *via* default parameters with 97% identity into operational taxonomic units (OTUs) (Edgar, 2013). The sequence with the highest frequency of occurrence in OTUs was selected as the representative sequence of OTUs for further annotation. OTUs abundance information was normalized using a standard of sequence number corresponding to the sample with the fewest sequences. Subsequent analysis of alpha diversity and beta diversity was performed based on this output normalized data. Chao1, ACE, Shannon and Simpson indices were calculated with the software package QIIME (version 1.9.1). Beta diversity analysis was performed using QIIME (version 1.9.1). The PCoA analyses were used to reveal the differences in the bacterial communities among the three groups (Minchin, 1987). The Adonis function of the R vegan package (version 2.15.3) was used to test the significance of separation by permutation multivariate analysis of variance (PERMANOVA) (Stat et al., 2013). Correlations were analyzed by using Spearman’s correlation in R software (version 2.15.3) with the R psych package and pheatmap for the heat map.

### Statistical analysis

All graphs were generated using GraphPad Prism version 8 and Adobe Illustrator 2022. All statistical analyses were performed using SPSS 26.0 software. The differences among groups were compared using One-way analysis of variance (ANOVA) and Bonferroni multiple comparison test. Data are represented as mean ± standard error. *P* < 0.05 indicates a significant difference. STAMP software (*t*-test) was used to analyze the differences of microbiota abundance between groups, and the Benjamini-Hochberg FDR multiple test correction method was used to control the false positive rate.

## Results

### Growth performance

As shown in Table 2, there was no significant difference in IBW, FBW, or ADFI (*P* > 0.05) among the three groups. ADG was increased in the L and H groups compared to the N group (*P* < 0.05). F/G was decreased in the L and H groups compared to the N group (*P* < 0.05).

**TABLE 2:** Effects of *Cyberlindnera jadinii* on the growth performance of growing raccoon dogs.

Items	Groups			*P*-value
	N	L	H	
IBW, kg	1.98 ± 0.05	1.96 ± 0.04	1.99 ± 0.11	0.968
FBW, kg	4.88 ± 0.35	5.47 ± 0.06	5.47 ± 0.25	0.152
ADG, g/day	43.10 ± 4.89^b^	60.46 ± 1.13^a^	60.00 ± 2.85^a^	0.002
ADFI, g/day	343.89 ± 6.64	334.69 ± 7.13	341.59 ± 6.75	0.620
F/G	8.09 ± 0.83^a^	5.53 ± 0.21^b^	5.82 ± 0.23^b^	0.008

IBW, Initial body weight; FBW, Final body weight; ADG, Average daily gain; ADFI, average daily feed intake; F/G, ratio of feed to gain. N group, 0 CFU/g *Cyberlindnera jadinii*; L group, 1 × 10^9^ CFU/g *Cyberlindnera jadinii*; H group, 5 × 10^9^ CFU/g *Cyberlindnera jadinii*. In the same row, values with no letter or the same letter superscripts indicate no significant difference (*P* > 0.05); different small letter superscripts indicate a significant difference (*P*< 0.05).

### Serum immune levels

As shown in Table 3, the serum IgA and IgG levels were increased in the L and H groups compared to the N group (*P* < 0.05). No significant differences in serum IgM levels were observed among the three groups (*P* > 0.05).

**TABLE 3:** Effects of *Cyberlindnera jadinii* on serum immune indices in growing raccoon dogs.

Items	Groups			*P*-value
	N	L	H	
IgA, μg/ml	36.28 ± 0.98^b^	41.72 ± 0.73^a^	41.00 ± 0.99^a^	0.001
IgG, μg/ml	324.844 ± 4.96^b^	368.31 ± 9.12^a^	367.20 ± 5.35^a^	0.001
IgM, μg/ml	19.34 ± 0.39	18.05 ± 0.45	19.43 ± 0.39	0.065

IgA, Immunoglobulin A; IgG, Immunoglobulin G; IgM, Immunoglobulin M; N group, 0 CFU/g *Cyberlindnera jadinii*; L group, 1 × 10^9^ CFU/g *Cyberlindnera jadinii*; H group, 5 × 10^9^ CFU/g *Cyberlindnera jadinii*. In the same row, values with no letter or the same letter superscripts indicate no significant difference (*P* > 0.05); different small letter superscripts indicate a significant difference (*P*< 0.05).

### Serum antioxidant capacity

As shown in Table 4, the activity of SOD in the L and H groups was increased compared to in the N group (*P* < 0.05), but no significant differences were observed between the L and H groups (*P* > 0.05). GSH-Px activity was increased in the L group compared with the N group (*P* < 0.05). No significant differences in serum T-AOC and MDA were observed among the three groups (*P* > 0.05).

**TABLE 4:** Effects of *Cyberlindnera jadinii* on serum antioxidant indices in growing raccoon dogs.

Items	Groups			*P*-value
	N	L	H	
GSH-Px, U/mL	364.63 ± 20.21^b^	469.51 ± 32.08^a^	393.90 ± 25.86^ab^	0.036
SOD, U/mL	28.56 ± 1.61^b^	36.85 ± 0.75^a^	36.79 ± 1.07^a^	< 0.001
T-AOC, U/mL	2.79 ± 0.29	2.98 ± 0.24	2.86 ± 0.25	0.871
MDA, nmol/mL	10.19 ± 1.20	11.90 ± 1.07	10.86 ± 0.85	0.524

GSH-Px, Glutathione peroxidase; SOD, Superoxide dismutase; T-AOC, Total antioxidant capacity; MDA, Malondialdehyde; N group, 0 CFU/g *Cyberlindnera jadinii*; L group, 1 × 10^9^ CFU/g *Cyberlindnera jadinii*; H group, 5 × 10^9^ CFU/g *Cyberlindnera jadinii*. In the same row, values with no letter or the same letter superscripts indicate no significant difference (*P* > 0.05); different small letter superscripts indicate a significant difference (*P* < 0.05).

### Summary of high-throughput sequencing and alpha diversity

**FIGURE 1:**
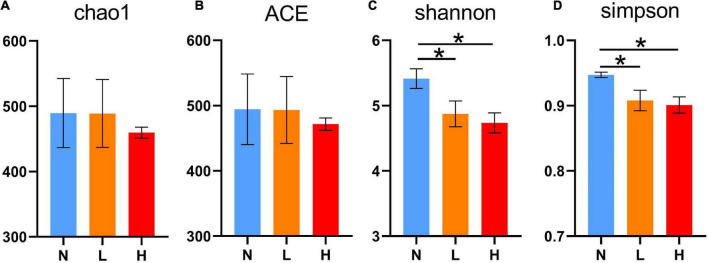
Comparisons of the alpha diversity indices of the raccoon dog gut microbiota among the three groups. Chao1 index **(A)**, ACE index **(B)**, Shannon index **(C)**, and Simpson index **(D)**. N group, 0 CFU/g *Cyberlindnera jadinii*; L group, 1 × 10^9^ CFU/g *Cyberlindnera jadinii*; H group, 5 × 10^9^ CFU/g *Cyberlindnera jadinii*. *P < 0.05.

The present study obtained a total of 1,436,812 16S rRNA gene sequences from three groups. After clustering at the 97% similarity level, sequences were assigned to 1,346 OTUs. Good’s coverage, ranging from 0.998 to 0.999, demonstrated an adequate sequencing depth for all samples. The number of OTUs was not significantly different among the three groups (*P* > 0.05, *N* = 456.17 ± 129.11, *L* = 447.33 ± 119.64 and *H* = 420.44 ± 97.52, respectively). As shown in Figure 1, the Shannon and Simpson index values in the N group were increased compared to the L and H groups (*P* < 0.05). There was no difference in the Chao1 and ACE indices among the three groups (*P* > 0.05).

### Composition and comparison of the gut microbiota in raccoon dogs

**FIGURE 2:**
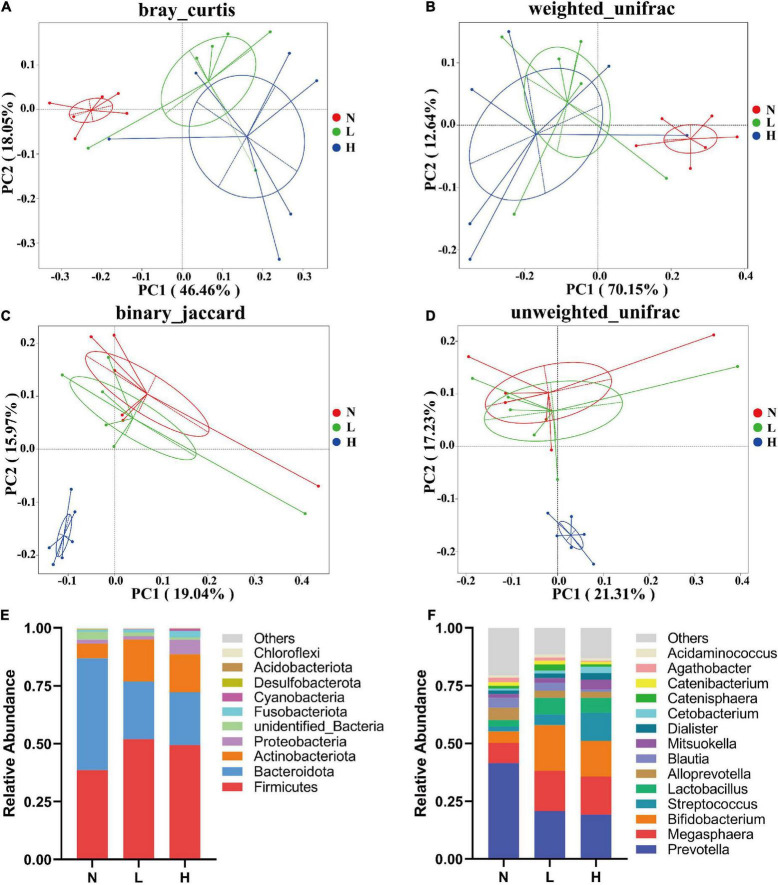
Composition and comparisons of the raccoon dog gut microbiota among the three groups. PCoA reveals the separation of the gut microbiota in the three groups based on the Bray–Curtis distance **(A)**, weighted UniFrac distance **(B)**, binary Jaccard distance **(C)** unweighted UniFrac distance **(D)**. Microbial composition in the gut of raccoon dogs from the N, L, and H groups at the phylum **(E)** and genus **(F)** levels. N group, 0 CFU/g *Cyberlindnera jadinii*; L group, 1 × 10^9^ CFU/g *Cyberlindnera jadinii*; H group, 5 × 10^9^ CFU/g *Cyberlindnera jadinii*.

PCoA was applied to examine differences in taxonomic community composition and structure in the gut of the raccoon dog. The PCoA based on the Bray–Curtis distance (Figure 2A) and weighted UniFrac distance (Figure 2B) showed that the N group was separated from the L and H groups [Table 5, Adonis: *P* < 0.05 (N vs. L, N vs. H)]. Whereas the PCoA based on the binary Jaccard distance (Figure 2C) and unweighted UniFrac distance (Figure 2D) showed that the H group was separated from the N and L groups [Table 5, Adonis: *P* < 0.05 (N vs. H, L vs. H)].

**TABLE 5:** Adonis analysis of the bacterial communities in the gut of growing raccoon dogs.

Group	Bray–Curtis		Weighted UniFrac		Binary Jaccard		Unweighted UniFrac	
	*R* ^2^	*P*-value	*R* ^2^	*P*-value	*R* ^2^	*P*-value	*R* ^2^	*P*-value
N vs. L	0.321	0.012	0.483	0.009	0.075	0.625	0.064	0.705
N vs. H	0.394	0.007	0.498	0.004	0.220	0.003	0.223	0.001
L vs. H	0.125	0.212	0.089	0.413	0.179	0.005	0.179	0.003

N group, 0 CFU/g *Cyberlindnera jadinii*; L group, 1 × 10^9^ CFU/g *Cyberlindnera jadinii*; H group, 5 × 10^9^ CFU/g *Cyberlindnera jadinii*.

At the phylum level, Bacteroidota (*N* = 48.17 ± 3.22%, *L* = 24.98 ± 4.16%), Firmicutes (*N* = 38.67 ± 2.76%, *L* = 51.89 ± 3.77%), Actinobacteriota (*N* = 6.33 ± 1.18%, *L* = 18.07 ± 4.82%), unidentified_Bacteria (*N* = 3.45 ± 3.40%, *L* = 1.64 ± 0.19%), and Proteobacteria (*N* = 1.73 ± 0.56%, *L* = 1.56 ± 0.47%) were the most abundant phyla in both the N and L groups, while Firmicutes (49.48 ± 3.96%), Bacteroidota (22.75 ± 6.67%), Actinobacteriota (16.42 ± 3.61%), Proteobacteria (6.20 ± 1.89%), and Fusobacteria (2.83 ± 1.81%) were the five most abundant phyla in the H group (Figure 2E). The ratio of Firmicutes to Bacteroidetes in the *Cyberlindnera jadinii* supplementation groups (*L* = 3.09 ± 0.60, *H* = 3.41 ± 0.90) were significantly increased compared with the N group (0.73 ± 0.07). At the genus level, *Prevotella* was the dominant genus in all three groups (*N* = 41.48 ± 2.73%, *L* = 20.80 ± 3.58%, *H* = 19.16 ± 5.72%). *Megasphaera* (8.74 ± 1.83%), *Alloprevotella* (5.26 ± 1.27%), *Bifidobacterium* (5.03 ± 1.07%), and *Blautia* (4.32 ± 0.75%) were the most abundant genera in the N group. *Bifidobacterium* (16.75 ± 4.73%), *Megasphaera* (17.31 ± 2.13%), *Lactobacillus* (4.08 ± 1.32%), and *Alloprevotella* (3.13 ± 0.54%) were the most abundant genera in the L group, and *Megasphaera* (14.88 ± 2.92%), *Bifidobacterium* (15.38 ± 3.68%), *Streptococcus* (12.28 ± 5.57%), and *Lactobacillus* (4.70 ± 0.40%) were the most abundant genera in the H group (Figure 2F).

**FIGURE 3:**
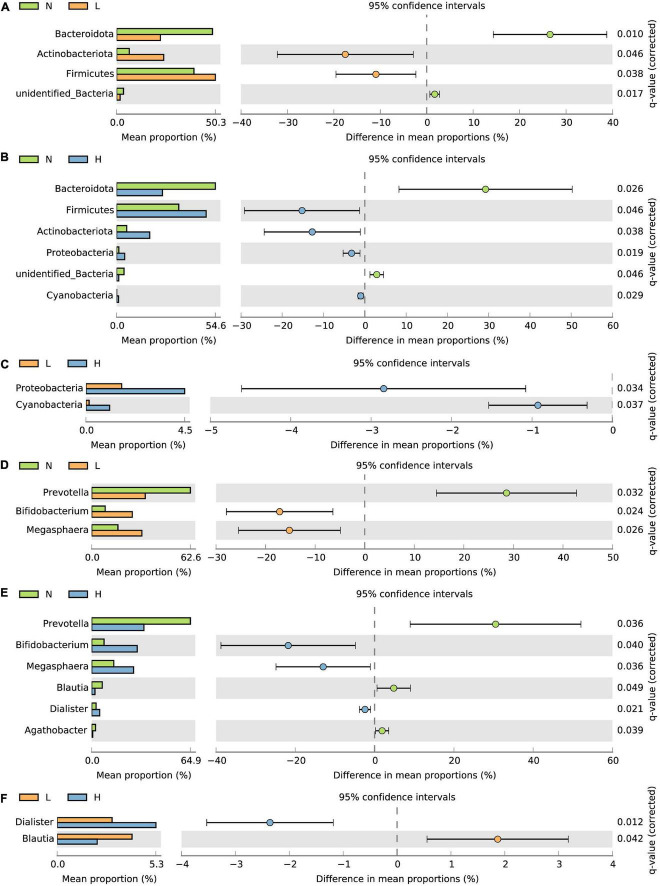
*T*-test bar plots showing differences in the relative abundance of bacteria at the phylum **(A–C)** and genus **(D–F)** levels. N group, 0 CFU/g *Cyberlindnera jadinii*; L group, 1 × 10^9^ CFU/g *Cyberlindnera jadinii*; H group, 5 × 10^9^ CFU/g *Cyberlindnera jadinii*.

Furthermore, we also compared the bacterial taxa among the three groups. The relative abundance of Firmicutes and Actinobacteriota was increased in the L and H groups compared to the N group, while the relative abundance of Bacteroidota and unidentified Bacteria were decreased in the L and H groups compared to the N group (*P* < 0.05) (Figures 3A,B). In addition, the relative abundance of Proteobacteria and Cyanobacteria in the H group was increased compared to the N and L groups (*P* < 0.05) (Figures 3B,C). At the genus level, the relative abundance of *Bifidobacterium* and *Megasphaera* was increased while the relative abundance of *Prevotella* was decreased in the L and H groups compared with the N group (*P* < 0.05) (Figures 3D,E). The relative abundance of *Agathobacter* was decreased in the H group compared with the N group (*P* < 0.05) (Figure 3E). The relative abundance of *Dialister* was increased where the relative abundance of *Blautia* decreased in the H group compared with the N and L groups (*P* < 0.05) (Figures 3E,F).

### Correlation between growth performance or serum markers and gut microbiota

**FIGURE 4:**
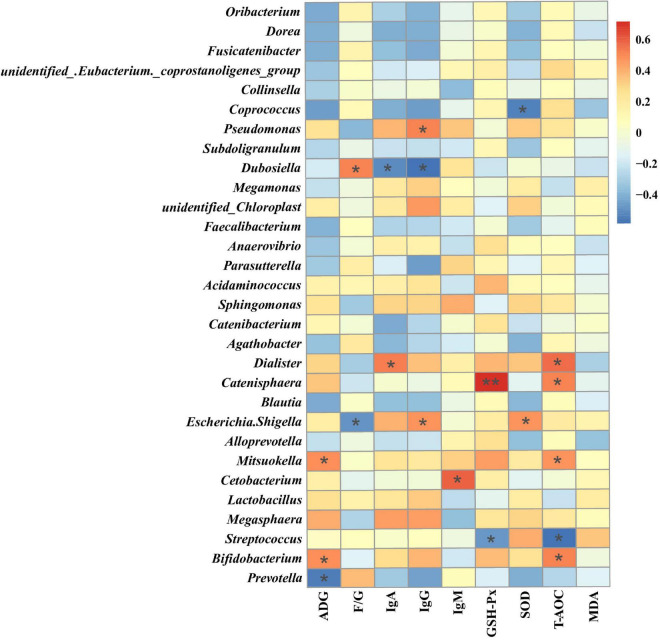
Heatmap of the Spearman rank correlation between the growth performance or serum markers and gut microbiota. ADG, Average daily gain; F/G, ratio of feed to gain; IgA, Immunoglobulin A; IgG, Immunoglobulin G; IgM, Immunoglobulin M; GSH-Px, Glutathione peroxidase; SOD, Superoxide dismutase; T-AOC, Total antioxidant capacity; MDA, Malondialdehyde. *P < *0.05*,**P < *0.01* (following the Spearman correlation analysis).

Spearman’s rank correlation analysis was performed to evaluate the potential relationship between alterations in gut microbiota composition and growth performance and serum markers of raccoon dogs. Sixteen genera, including *Prevotella*, *Bifidobacterium* and *Dialister*, showed significant correlations with the serum markers (Figure 4). The genus *Prevotella* showed a significant negative correlation with ADG (*P* < 0.05), the genus *Bifidobacterium* showed a significant positive correlation with ADG and serum T-AOC (*P* < 0.05), and *Dialister* showed a significant positive correlation with serum IgA and T-AOC (*P* < 0.05) (Figure 4).

## Discussion

Previous studies have suggested that *Cyberlindnera jadinii* can improve the growth performance of weaned piglets (Yang et al., 2021). Our results showed that *Cyberlindnera jadinii* increased ADG and decreased the F/G in raccoon dogs. Additionally, the dietary supplementation of *Cyberlindnera jadinii* at 1 × 10^9^ CFU/g resulted in the highest ADG and the lowest F/G. This may be because yeast can bind metal ions in the environment and permanently integrate into their cellular structure. Due to active transport mechanisms, absorbed elements can be transferred to the inside of the cell and accumulate in the form of metal. This can lead to the formation of stable complexes with proteins. Due to these connections, nutrient elements are more readily collected, transported, and absorbed in the digestive tracts of animals (Kieliszek et al., 2017).

Serum immunoglobulin can be used as a parameter reflecting the immune state of animals. Research has shown that diets supplemented of *Saccharomyces cerevisiae* increased the level of serum IgA in piglets (Trckova et al., 2014; Jiang et al., 2015). In addition, diets supplemented with *Saccharomyces cerevisiae* and yeast culture increased the levels of IgA, IgG and IgM in lambs during the growing period (Mahmoud et al., 2020). Our results showed that *Cyberlindnera jadinii* improved the levels of IgA and IgG in raccoon dogs. The changes in serum immunoglobulins observed in our studies may be related to β-glucans found in yeast cell walls, which have immunomodulatory properties (Raghebian et al., 2016). β-glucans increase the host immune defense by activating the complement system and enhancing macrophage and natural killer cell function (Akramiene et al., 2007). In addition, oxidative stress is a common condition which can produce variety of oxygen free radicals. An excess of oxygen free radicals can damage proteins, nucleic acids, and other biological macromolecules, thus causing tissue damage and tissue mitochondrial damage (Bai et al., 2018). In our study, *Cyberlindnera jadinii* dietary supplementation increased serum SOD and GSH-Px activities. An increase in SOD activity is an element of the natural antioxidant defense system (Yousefi et al., 2020), and the increased GSH-Px activity may be due to the presence of glutathione-bound selenium compounds in yeasts. Yeasts are characterized by a relatively high capacity to accumulate selenium (Kieliszek et al., 2016, 2017). Selenium is an essential constituent of the GSH-Px, and GSH-Px catalyzes the reduction of hydrogen peroxide by glutathione (Patching and Gardiner, 1999). The GSH and GSH-Px enzymes relieve oxidative damage by eliminating excessive free radicals (Bai et al., 2018). The results of the current study showed that *Cyberlindnera jadinii* supplementation improved the immune state and antioxidant activity in raccoon dogs.

The intestinal microbiota is closely related to the growth and development of animals. The gut microbiota of the raccoon dogs was dominated by sequences representative of Firmicutes, Bacteroidetes, Actinobacteria, and Proteobacteria. The phyla Firmicutes, Proteobacteria, Bacteroidetes, and Actinobacteria are also widely present in the gastrointestinal tracts of other carnivore species such as mink, Eurasian otters, leopard cats, blue fox and silver fox (An et al., 2017; Peng et al., 2019; Liu et al., 2020; Nan et al., 2021). Our results showed that at the phylum level, *Cyberlindnera jadinii* supplementation increased the relative abundance of Firmicutes and Actinobacteria but decreased the relative abundance of Bacteroidetes. In addition, 5 × 10^9^ CFU/g *Cyberlindnera jadinii* also improved the relative abundance of Proteobacteria and Cyanobacteria. Moreover, the Firmicutes/Bacteroidetes ratio (F/B ratio) was increased in the *Cyberlindnera jadinii* supplementation groups. The Firmicutes phyla contain genes that are related to energy metabolism and the decomposition of substances (Kaakoush, 2015; Zhang et al., 2019), and Bacteroidetes are associated with the degradation of proteins and carbohydrates (Thomas et al., 2011; Waite and Taylor, 2014). Previous studies found that the F/B ratio is proportional to body weight (Singh et al., 2013). A high F/B ratio is beneficial for gut microbiota-mediated energy harvesting in animals (Li et al., 2016), which assists with the maintenance of metabolic balance and better growth performance (Ley et al., 2006; Murphy et al., 2010). Therefore, this study suggests that the gut microbiota following *Cyberlindnera jadinii* supplementation had a strong effect on energy metabolism and the decomposition of substances. This strong ability to obtain energy may also be one reason for their strong growth performance. In addition, the composition of the gut microbiota is influenced by endogenous and environmental factors (Laparra and Sanz, 2010). In general, the diet is considered a major driver of changes in gut microbial diversity, which may affect its functional relationship with the host (Ley et al., 2008; Laparra and Sanz, 2010). However, multiple dietary components can interact non-additively to influence gut microbial diversity (Bolnick et al., 2014). In the current study, the *Cyberlindnera jadinii* supplementation decreased the alpha diversity of the gut microbiota as shown by the Shannon and Simpson indices. It may be that *Cyberlindnera jadinii* supplementation has indirect effects on raccoon dog physiology and immunity, which may regulate the diversity of the gut microbiota. Alternatively, *Cyberlindnera jadinii* supplementation might increase the activity of certain bacteria that affect the presence or growth of certain gut microbiota. For example, Actinobacteria can produce secondary metabolites, many of which have antibacterial and antifungal properties (Ul-Hassan and Wellington, 2009). *Cyanobacteria* is a diverse source of compounds with antimicrobial activity (Swain et al., 2017). Consequently, the alpha diversity of the gut microbiota may decrease with *Cyberlindnera jadinii* supplementation. Given that an imbalanced gut microbiota often arises from a sustained increase in abundance of the phylum Proteobacteria, the natural gut microbiota normally contains only a minor proportion of this phylum (Shin et al., 2015). The anaerobic Proteobacteria are usually associated with an impaired microbiota, or dysbiosis (Litvak et al., 2017). Thus, dietary supplementation of 1 × 10^9^ CFU/g *Cyberlindnera jadinii* may be the most beneficial dose for the balance of intestinal microbiota in growing raccoon dogs.

Our results showed that at the genus level, *Cyberlindnera jadinii* increased the relative abundance of *Megasphaera* and *Bifidobacterium* and decreased the relative abundance of *Prevotella*. Some strains of *Megasphaera* can produce several short-chain fatty acids (SCFAs) such as acetate, propionate, butyrate, and valerate (Yoshikawa et al., 2018). These SCFAs may provide energy sources for animal growth. The increase of *Bifidobacterium* may be due to the presence of bifidus factors in yeast that promote the growth of *Bifidobacterium* (Ghoddusi and Tamime, 2014). Spearman correlation analysis also showed that ADG exhibited a significant, positive correlation with the relative abundance of *Bifidobacterium*. From a metabolic point of view, Bifidobacteria use the fructose 6-phosphate pathway for the metabolism of glucose and lactose, which can also provide energy sources for animal growth (González-Rodríguez et al., 2013; Hidalgo-Cantabrana et al., 2017). Moreover, the abundance of *Prevotella* is negatively correlated with weight changes (Christensen et al., 2019), which provids further support for the increase of ADG in raccoon dogs. Some strains of *Prevotella*, identified as active microbes, are associated with plant-rich diets and can express various genes encoding carbohydrate-degrading enzymes (Dai et al., 2015). Our results showed that the gut microbiota following *Cyberlindnera jadinii* supplementation had a lower abundance of cellulose-degrading bacteria, which may be more suitable to the dietary habits of carnivores. In addition, adding 5 × 10^9^ CFU/g *Cyberlindnera jadinii* in the feed also increased the relative abundance of *Dialister* and decreased the relative abundance of *Blautia* and *Agathobacter*. *Dialister*, a potential microbial marker of disease activity, has been shown to exhibit a positive correlation with disease activity (Tito et al., 2017). Moreover, we observed a significant positive correlation between the abundance of the *Dialister* and serum IgA concentration, as shown by spearman correlation analysis. This may be an immune defense mechanism of the host. *Blautia* is a genus of anaerobic bacteria with probiotic characteristics that occur widely in the feces and intestines of mammals. *Blautia* has been shown to play a role in metabolic diseases, inflammatory diseases, and biotransformation (Liu et al., 2021). The main fermentation product of *Agathobacter* is butyrate (Hua et al., 2020). Butyrate is a short-chain fatty acid that plays an important role in gut physiology. Butyrate can enhance the barrier function of the gut and represses inflammatory responses through inhibition of NF-kB activation (Macfarlane and Macfarlane, 2011). Results of the current study suggest that adding 5 × 10^9^ CFU/g *Cyberlindnera jadinii* may decrease the abundance of beneficial bacteria and increase the abundance of microbiota related to intestinal inflammation. Therefore, *Cyberlindnera jadinii* may play an active role in growth performance by regulating intestinal microbiota, and this effect was greater when the addition level was 1 × 10^9^ CFU/g.

## Conclusion

The present research demonstrated that dietary *Cyberlindnera jadinii* supplementation improved growth performance, serum antioxidant capacity and immunity, and intestinal microbiota in growing raccoon dogs. Among the concentrations tested, 1 × 10^9^ CFU/g was the most effective level of supplementation. Thus, *Cyberlindnera jadinii* has potential as an efficient antibiotic alternative in raccoon dog feed.

## Data availability statement

The datasets presented in this study can be found in online repositories. The names of the repository/repositories and accession number(s) can be found below: NCBI BioProject – PRJNA853184.

## Ethics statement

The animal study was reviewed and approved by the Animal Care Committee of Chifeng University and conducted in strict compliance with the Committee’s guidelines on animal care. Written informed consent was obtained from the owners for the participation of their animals in this study.

## Author contributions

DZ: conceptualization, formal analysis, and writing—original draft. HL: funding acquisition, methodology, project administration, and writing—review and editing. HZ: methodology, validation, and resources. KL and XZ: data curation, investigation, and supervision. QL, YW, TZ, and QZ: data curation and sample collection. All authors have read and approved the final manuscript.
